# Clinical and Genomic Features of Patients with Renal Cell Carcinoma and Advanced Chronic Kidney Disease: Analysis of a Multi-Institutional Database

**DOI:** 10.3390/cancers16101920

**Published:** 2024-05-18

**Authors:** Corbin J. Eule, Junxiao Hu, Dale Hedges, Alkesh Jani, Thomas Pshak, Brandon J. Manley, Alejandro Sanchez, Robert Dreicer, Zin W. Myint, Yousef Zakharia, Elaine T. Lam

**Affiliations:** 1 Division of Medical Oncology, Department of Medicine, University of Colorado Anschutz Medical Campus, Aurora, CO 80045, USA; 2 Biostatistics Core, University of Colorado Cancer Center, Aurora, CO 80045, USA; 3 Aster Insights, Hudson, NY 13610, USA; 4 Division of Nephrology, Department of Medicine, University of Colorado Anschutz Medical Campus, Aurora, CO 80045, USA; alkesh.jani@cuanschutz.edu; 5 Division of Urology, Department of Surgery, University of Colorado Anschutz Medical Campus, Aurora, CO 80045, USA; 6 Department of Genitourinary Oncology, Moffitt Cancer Center, Tampa, FL 33612, USA; brandon.manley@moffitt.org; 7 Division of Urology, Department of Surgery, Huntsman Cancer Institute, University of Utah, Salt Lake City, UT 84112, USA; 8 Division of Medical Oncology, Department of Medicine, University of Virginia Comprehensive Cancer Center, Charlottesville, VA 22908, USA; 9 Division of Medical Oncology, Department of Internal Medicine, Markey Cancer Center, University of Kentucky, Lexington, KY 40506, USA; 10 Division of Hematology, Oncology, and Blood and Bone Marrow Transplantation, Department of Internal Medicine, Holden Comprehensive Cancer Center, University of Iowa, Iowa City, IA 52242, USA

**Keywords:** renal cell carcinoma, chronic kidney disease, genomics, BAP1, mutation

## Abstract

**Simple Summary:**

Despite the increased risk of developing renal cell carcinoma (RCC) in patients with advanced chronic kidney disease (ACKD), little is known about the patient clinical characteristics and genetic mutations found in these RCC tumors. Using a multi-institutional research network, this study compiled clinical records and somatic tumor whole exome sequencing data of 296 adult patients with RCC, 61 of whom had ACKD. Patients with RCC and ACKD were more likely to be male, present with earlier stage RCC at diagnosis, and have lower rates of *BAP1* mutations. Median overall survival was not reached in either group over a median follow-up of 31.3 months. These findings suggest RCC in patients with ACKD develops via a *BAP1*-independent mutational driver and further support *BAP1* loss as a marker of disease aggressiveness.

**Abstract:**

Background: Patients with advanced chronic kidney disease (ACKD) are at an increased risk of developing renal cell carcinoma (RCC), but molecular alterations in RCC specimens arising from ACKD and overall survival (OS) in affected patients are not well defined. Patients and Methods: Using the Oncology Research Information Exchange Network (ORIEN) Total Cancer Care^®^ protocol, 296 consented adult patients with RCC and somatic tumor whole exome sequencing were included. Patients with ACKD were defined as those with serum creatinine ≥1.5 mg/dL prior to RCC diagnosis. Results: Of 296 patients with RCC, 61 met the criteria for ACKD. The most common somatic mutations in the overall cohort were in *VHL* (126, 42.6%), *PBRM1* (102, 34.5%), and *SETD2* (54, 18.2%). *BAP1* had a decreased mutational frequency in RCC specimens from patients without ACKD as compared to those with ACKD (10.6% versus 1.6%), but this was not statistically significant in univariable (OR 0.14, *p* = 0.056) or multivariable (OR 0.15, *p* = 0.067) analysis. Median OS was not reached in either cohort. Conclusions: Using the clinicogenomic ORIEN database, our study found lower rates of *BAP1* mutations in RCC specimens from patients with ACKD, which may reflect a *BAP1*-independent mutational driver of RCC in patients with ACKD.

## 1. Background

Renal cell carcinoma (RCC) is characterized by diverse histopathologic subtypes, contributing to its clinical heterogeneity and the therapeutic challenges facing this disease. The most common RCC subtype is clear cell RCC (approximately 80%) followed by papillary (15%) and chromophobe (5–10%) RCC [1,2,3].

End-stage renal disease (ESRD), kidney transplant, and impaired kidney graft function are associated with an increased risk of developing RCC compared to the general population [4,5]. Chronic kidney disease (CKD) and acquired cystic kidney disease have also been shown to increase RCC risk [6,7,8]. In a large, population-based cohort study using national dialysis, transplant, and cancer registries in Australia and New Zealand, Vajdic and colleagues demonstrated a standard incidence ratio of 13.2 (95% confidence interval [CI] 11.4, 15.1) for RCC in patients with CKD up to 5 years prior to initiation of dialysis [9]. Despite this demonstrated epidemiologic connection between CKD and RCC, the underlying molecular etiologies are not well elucidated [7,10].

The Cancer Genome Atlas (TCGA) Research Network identified multiple significantly mutated genes (SMGs) from analysis of over 500 clear cell RCC and papillary RCC tumors [11,12]. In clear cell RCC, the most common mutation affects the von Hippel Lindau (*VHL*) tumor suppressor gene involved in cellular oxygen sensing and protein polybromo 1 (*PBRM1*) gene, which controls the maintenance of chromatin [12]. Mutations inactivating the BRCA1-associated protein (*BAP1*), a nuclear deubiquitinase, is associated with more clinically aggressive variants of clear cell RCC [13]. Mutations in the chromatin remodeling genes *PBRM1, BAP1,* and SET domain containing 2 (*SETD2*) have been frequently implicated in sporadic cases of both clear cell RCC and type 2 papillary RCC [11,12]. However, hereditary cases of papillary RCC are associated with MET pathway dysregulation (type 1) or pathogenic variants in the fumarate hydratase (*FH*) gene (type 2) [14,15].

Compared to sporadic cases of clear cell RCC, clear cell RCC occurring in patients with ESRD may have lower rates of chromosome 3p loss, which includes *VHL* [16,17]. However, studies of molecular alterations in RCC and ESRD have primarily been limited to chromosomal analysis and have not included patients with RCC and non-ESRD renal dysfunction [18]. Interestingly, patients with RCC and ESRD have been shown to have lower rates of metastasis and longer cancer-specific survival compared to the general population [19]. Whether this trend also exists for patients with RCC and CKD is unknown.

This study seeks to contribute to a deeper understanding of the clinical characteristics and molecular mechanisms underlining RCC in the presence of advanced CKD (ACKD) and its effect on patient outcomes.

## 2. Methods

### 2.1. Data Source

The Oncology Research Information Exchange Network (ORIEN) is an alliance of 18 U.S. cancer centers established in 2014. All ORIEN alliance members utilize a standard Total Cancer Care^®^ (TCC) protocol. As part of the TCC study and ORIEN Avatar program, participants agree to have their clinical data followed over time, to undergo germline and tumor sequencing, and to be contacted in the future by their provider if an appropriate clinical trial or other study becomes available [20]. Aster Insights, the commercial and operational partner of ORIEN, harmonizes all abstracted clinical data elements and molecular sequencing files into a standardized, structured format to enable aggregation of de-identified data for sharing across the network.

### 2.2. Patient Population

Patients aged 18 years or older with a histologic diagnosis of RCC and somatic tumor WES data available in the multi-institutional ORIEN database were included. Patients with ACKD were defined as those with a serum creatinine value of ≥1.5 mg/dL prior to a diagnosis of RCC. Both demographic and clinical information of patients were extracted from ORIEN and stratified based on the presence or absence of ACKD.

### 2.3. Sequencing Methods and Analysis

ORIEN Avatar specimens underwent nucleic acid extraction and sequencing at HudsonAlpha (Huntsville, AL, USA) or Fulgent Genetics (Temple City, CA, USA). For frozen and optimal cutting temperature (OCT) tissue DNA extraction, Qiagen QIASymphony DNA purification was performed, generating 213 bp average insert size. For FFPE tissue, the Covaris Ultrasonication FFPE DNA/RNA kit was utilized to extract DNA and RNA, generating a 165b bp average insert size. For DNA sequencing, preparation of Aster Insights Whole Exome Sequencing (WES) libraries involved hybrid capture using an enhanced IDT WES kit (38.7 Mb) with additional custom designed probes for double coverage of 440 cancer genes. Library hybridization was performed at either single or 8-plex and sequenced on an Illumina NovaSeq 6000 instrument (Illumina, San Diego, CA, USA) generating 100 bp paired reads. WES was performed on tumor/normal matched samples with the normal sample covered at 100× and the tumor covered at 300× (additional 440 cancer genes were covered at double coverage; 200× for normal and 600× for tumor). Both tumor/normal concordance and gender identity QC checks were performed. The minimum threshold for hybrid selection was >80% of bases with >100× fold coverage for the tumor and >50× fold coverage for the normal sample.

Mutation targets were determined by the significantly mutated genes (SMGs) identified by TCGA (Supplementary Table S1) [12,13,14,15]. Mutations must be somatic, have intersected the region of a SMG, passed all standard quality filters applied by Sentieon and Aster Insights, including a panel of normal filtration set to a mean allele frequency (MAF) > 1% for common artifacts and polymorphisms, have a minimum of 5 reads, and are non-silent protein-altering mutations. Somatic mutations were stratified by ACKD status.

### 2.4. Statistical Analysis

Summary statistics were reported for clinical and demographic characteristics. Continuous variables were presented with median and IQR. The categorical variables were presented with frequency values and percentages. The Fisher’s exact test, or non-parametric test, was used for the comparison of continuous and categorical variables by ACKD presence, as shown in Table 1 and Table 2.

The survival probability was calculated by the Kaplan–Meier method. The Kaplan–Meier survival curve and the median survival time were reported with the corresponding 2-sided 95% confidence interval (CI) if feasible. The log-rank test was conducted to compare the overall survival (OS) stratified by ACKD presence. The Cox proportional hazard model was used to compare the OS by ACKD presence and mutation status while adjusting for important covariates. Logistic regressions were conducted to evaluate the association of mutation status to ACKD presence while adjusting for important covariates. Univariate analysis using the Cox proportional hazard model or logistic regression model were conducted to evaluate the association of the covariates and the primary outcomes. The selection of covariates in multivariable analysis was based on the univariate analysis results and previous literature reviews. The covariates considered included age, sex, histology, year of diagnosis, and stage.

The statistical significance level was set to 0.05. All statistical analyses were conducted using R version 4.1.0, R Core Team (2021).

## 3. Results

A total of 296 patients diagnosed with RCC and consented to the TCC protocol from the participating ORIEN members were included in this study (Table 1). Of these, 61 patients (20.6%) had ACKD. The patients with ACKD were diagnosed at a median age of 63.0 years compared to 61.2 years for patients without ACKD (*p* = 0.045). The overall cohort was predominantly male (191 patients, 64.5%), with an even greater proportion of male patients having ACKD (49, 80.3%; *p* = 0.006). Patients were most often white (260, 87.8%) with clear cell histology (191, 64.5%). Papillary RCC histology was approximately twice as common in patients with ACKD (18.0% versus 8.9%). Most patients were diagnosed with stage I (146, 49.3%) or stage III (100, 33.8%) disease.

The most common somatic mutations in the overall cohort were in *VHL* (126, 42.6%), *PBRM1* (102, 34.5%), and *SETD2* (54, 18.2%) (Table 2). Among 29 SMGs in RCC, only *BAP1* showed a statistically significant increase in the mutational frequency from RCC specimens of patients without ACKD as compared to those with ACKD (10.6% versus 1.6%, respectively; *p* = 0.050). Somatic mutational rates of *BAP1* were primarily reported in patients with clear cell RCC histology (20/156 patients, 12.8% without ACKD and 1/35 patients, 2.9% with ACKD). However, when the analysis was limited to patients with clear cell RCC only, this difference was not statistically significant (*p* = 0.132) (Supplementary Table S2). *TP53* mutations were also less common in RCC specimens of all patients without ACKD versus patients with ACKD (3.4% versus 9.8%, respectively), but this was not statistically significant (*p* = 0.077). This difference was similarly noted in patients with clear cell RCC (4/156 patients, 2.6% without ACKD versus 3/35, 8.6% with ACKD; *p* = 0.117).

While *BAP1* mutations were less frequent in patients with ACKD (univariable OR 0.14, multivariable OR 0.15), this finding was ultimately not statistically significant (*p* = 0.056 and *p* = 0.067, respectively) (Table 3). On both univariable and multivariable analysis, *BAP1* was positively correlated with stage III (univariable OR 9.08, *p* = 0.001; multivariable OR 8.97, *p* = 0.001) and stage IV (univariable OR 7.09, *p* = 0.014; multivariable OR 6.32, *p* = 0.021) disease. Notably, there was no statistically significant correlation with *BAP1* and age at diagnosis, sex, or RCC histology.

Mutations in *VHL*, despite their high prevalence, had no statistically significant correlation to the clinical features of patients with RCC in univariable or multivariable analysis (Supplementary Table S3). Additionally, there was no difference in rates of *VHL* mutations in clear cell RCC specimens from patients without ACKD compared to those with ACKD (52.6% versus 45.7%, *p* = 0.575) (Supplementary Table S2).

**Figure 1:**
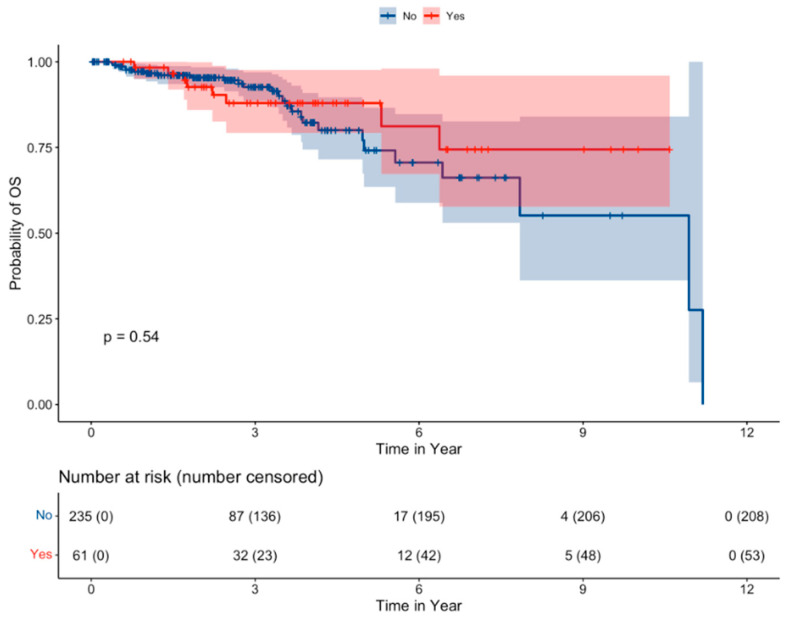
Kaplan–Meier survival curve of patients with RCC stratified by the presence of ACKD.

Median OS was not reached for patients with or without ACKD over a median follow-up of 31.3 months (IQR 18.7–48.9 months) (Figure 1). ACKD in patients with RCC did not lead to differences in OS (*p* = 0.54), including univariable (0.78 [95% CI 0.35–1.74], *p* = 0.541) and multivariable (1.08 [95% CI 0.45–2.59], *p* = 0.866) analysis. Analysis of age at diagnosis, sex, race, RCC histology, year of diagnosis category, and stage revealed that only stage IV disease was meaningfully associated with decreased OS (univariable HR 4.65 [95% CI 2.01–10.77], *p* < 0.001).

## 4. Discussion

While ACKD impacts the clinical management of patients with RCC, its role in RCC tumorigenesis is less understood. Using the multi-institutional ORIEN database, this study compiled a cohort of patients with RCC and corresponding tumor WES data to evaluate the somatic mutational differences in patients with or without preceding ACKD. While RCC in ESRD has been shown to be less clinically aggressive, there was no difference in OS for patients with ACKD versus patients without ACKD in this study [19]. Nonetheless, patients with ACKD were less likely to have stage III or IV disease at diagnosis. This finding raises the possibilities that our study was underpowered to detect a meaningful difference in survival or survival may have been impacted by the effect of the underlying CKD comorbidity rather than a result of RCC. Additionally, a median follow-up of 31.3 months may be an insufficient duration to detect a survival difference even in metastatic RCC. For example, updated survival and efficacy data from CheckMate 214 demonstrated a median OS of 55.7 months in the ipilimumab plus nivolumab cohort and 38.4 months in the sunitnib cohort of patients with advanced RCC [21].

The patients in our study were predominantly men, but this was most pronounced in patients with ACKD (80.3% men). While the rates of RCC are higher in men in the United States, CKD is slightly more common in women, in contrast to the ORIEN cohort [22,23]. However, men with CKD have higher risk of eGFR decline, progression to CKD stage 5, and ESRD as compared to women with CKD [24]. This may have contributed to a greater proportion of men meeting the definition for the ACKD cohort in our study. Furthermore, the biologic factors driving sex-related differences for CKD progression are unknown [24]. Given the unexpected male predominance of RCC in ACKD, this could suggest that eGFR decline may also be an independent risk factor for RCC tumorigenesis.

The rates of papillary RCC, which is known to occur more often in the context of ACKD, were higher in the ORIEN cohort with ACKD (18.0% versus 8.9%) [9]. As the WES data were restricted to somatic mutations, a fraction of germline mutations may not have been detected due to somatic and germline DNA heterogeneity. *MET* mutations such as those found in hereditary type 1 papillary RCC were uncommon, making up 2.7% of the overall cohort. Similarly, the *FH* mutation found in type 2 papillary RCC associated with hereditary leiomyomatosis and renal cell carcinoma (HLRCC) was found in only one patient [25]. *SETD2* mutations, an SMG in both papillary RCC and ccRCC, were present in none of the papillary cases [11].

The rates of mutations in *VHL*, *PBRM1*, *BAP1*, and *SETD2* in the overall ORIEN cohort were comparable to those found in TCGA for clear cell RCC [11,12]. In clear cell RCC, the loss of chromosome 3p, the location of these four tumor suppressor genes, is often the initial step in tumorgenesis [26]. The inactivation of the second allele of *VHL* is an essential next step that precipitates clonal and subclonal expansion, ultimately leading to the development of clinical clear cell RCC [26]. As such, chromosome 3p loss and more specifically *VHL* inactivation have emerged as therapeutic targets in clear cell RCC [27]. *VHL* inactivation leads to the accumulation of hypoxia-inducible factor 2α (HIF2α) and subsequent upregulation of vascular endothelial growth factor (VEGF) [27]. Consistent with its central role in clear cell RCC development, *VHL* mutations were common in the ORIEN cohort, but not found to vary significantly between patients with and without ACKD, both in the overall cohort and clear cell RCC subgroup. *VHL* is a small gene with three exons and is CG rich, which may result in undercalled mutations. Additionally, *VHL* function can be altered through chromosomal loss or methylation, which may not be detected in this analysis [28].

The chromatin remodeling genes *PBRM1*, *BAP1*, and *SETD2* on chromosome 3p have also been implicated in papillary RCC and clear cell RCC development in conjunction with *VHL* inactivation [11,29,30]. *BAP1* loss, in particular, has been associated with a high tumor grade and worse survival outcomes for patients with clear cell RCC [31,32]. Notably, rates of *BAP1* mutations in patients with RCC and ACKD were low at 1.6% relative to patients without ACKD in the ORIEN cohort and historical controls (estimated prevalence of approximately 15% in clear cell RCC and papillary RCC) [31,32]. While decreased *BAP1* mutational frequency in ACKD was not statistically significant in univariable and multivariable analysis, this finding could suggest that RCC in patients with ACKD is more likely to arise from a *BAP1*-independent process. Furthermore, *BAP1* inactivation in patients without ACKD may contribute to more clinically aggressive disease manifested by higher stages at diagnosis.

When *BAP1* loss occurs, this mutation is likely the driver in sporadic cases of clear cell RCC due to its association with low intratumoral heterogeneity and high tumor grade and proliferation [33]. In contrast, RCC tumorigenesis in ACKD likely originates from cellular damage and aberrant repair pathways in response to uremic toxicity and oxidative stress [10]. Ischemic kidney injury has been shown to upregulate *NOTCH1*, which regulates cell proliferation and regeneration [34]. *NOTCH1* overexpression was found to have a time-dependent onset in clonal papillary tumors from a mouse model and induced tumor-like growth in human renal progenitor cells. In light of recent findings on cancer stem cells (CSCs) in RCC, which outline the role of key signaling pathways such as Notch and Wnt in CSC regulation, it is conceivable that similar pathways altered in this study’s patients with RCC and ACKD may support the CSC hypothesis [35]. This overlap suggests that CSCs could play a significant role in the pathogenesis and progression of RCC in ACKD.

Alternate pathways, such as those involving mucin short variant S1 (*MUC1*), have been linked to chronic inflammation and are frequently overexpressed in RCC [36]. *MUC1* is transiently elevated during acute kidney injury to promote recovery, but prolonged overexpression can lead to CKD [36,37]. Given the role of *MUC1* in critical oncogenic pathways such as cell proliferation, metabolic reprogramming, and angiogenesis, this pathway provides a potential link to our findings. The interaction of *MUC1* with these pathways could be particularly impactful in the context of RCC with ACKD, suggesting a potential target for therapeutic intervention and a biomarker for assessing disease progression and treatment efficacy.

This study illustrates the need for a better understanding of RCC tumorigenesis in ACKD, as it has the potential to reveal pathogenic mechanisms of RCC development beyond mutations in *VHL*, *PBRM1*, and *BAP1*. Despite the availability of next generation sequencing in clinical practice, the molecular characterization of RCC is not routinely used by oncologists in treatment decision making. While genomic testing is often used to predict outcomes in tumor types such as prostate cancer, genomic risk stratification remains an unmet need in RCC [38]. Clinical criteria have been used to predict clear cell RCC in patients with an increased risk of recurrence after surgery who may benefit from adjuvant pembrolizumab, as shown in the KEYNOTE-564 trial [39]. However, genomic risk profiling in RCC may add further granularity to patient risk categorization and in turn, prevent over- or undertreatment. In particular, RCC in ACKD and ESRD may increase our understanding of low-risk genomic features in RCC given its association with lower metastatic potential and increased cancer-specific survival [19].

This study was limited by its small sample size. Despite higher rates of ACKD in black patients, 85.2% of patients with ACKD in the study were white. Additionally, missing data on RCC histological subtypes as well as the etiology and duration of ACKD may have led to the over or underestimation of their influence on the analysis.

## 5. Conclusions

There is a dearth of knowledge about the genomic association between ACKD and RCC. Using the clinicogenomic ORIEN database, our study examined the mutational frequency of 29 SMGs in RCC in patients with and without ACKD. Lower rates of *BAP1* mutations in RCC specimens from patients with ACKD point to a potential *BAP1*-independent mutational driver of RCC in patients with ACKD. The association between *BAP1* loss and presence of metastases further supports this mutation as a marker of disease aggressiveness and underscores the clinical importance of *BAP1* in future therapeutic development.

## Figures and Tables

**Table 1:** Baseline characteristics of patients with RCC in the ORIEN database stratified by presence of ACKD.

		No ACKD *n* (%)	ACKD *n* (%)	Total *n* (%)	*p* Value
Total Patients		235 (79.4)	61 (20.6)	296	
Age at Diagnosis	Median (IQR)	61.2 (52.3 to 67.0)	63.0 (56.5 to 72.5)		0.045
Sex	Female	93 (39.6)	12 (19.7)	105 (35.5)	0.006
	Male	142 (60.4)	49 (80.3)	191 (64.5)	
Race and Ethnicity	American Indian or Alaska Native	4 (1.7)	1 (1.6)	5 (1.7)	0.722
	Asian	6 (2.6)	1 (1.6)	7 (2.4)	
	Black or African American	11 (4.7)	5 (8.2)	16 (5.4)	
	Hispanic/Latino	25	2		
	Other	1 (0.0)	0 (0.0)	8 (2.7)	
	Non-Hispanic/Latino White	188 (80.0)	52 (85.2)	260 (87.8)	
Histology	Clear Cell	156 (66.4)	35 (57.4)	191 (64.5)	0.184
	Papillary	21 (8.9)	11 (18.0)	32 (10.8)	
	Chromophobe	17 (7.2)	3 (4.9)	20 (6.8)	
	NOS *	41 (17.4)	12 (19.7)	53 (17.9)	
Creatinine Prior to Diagnosis	Median mg/dL (IQR)	0.9 (0.8 to 1.1)	1.5 (1.3 to 1.7)		<0.001
Year of Diagnosis	Median (IQR)	2018.0 (2017.0 to 2019.0)	2017.0 (2015.0 to 2019.0)		0.091
Stage	I	105 (44.7)	31 (50.8)	146 (49.3)	0.402
	II	23 (9.8)	8 (13.1)	28 (9.5)	
	III	81 (34.5)	19 (31.1)	100 (33.8)	
	IV	26 (11.1)	3 (4.9)	29 (9.8)	

* Not otherwise specified.

**Table 2:** Somatic mutation rates in RCC specimens stratified by presence of ACKD.

Mutated Gene	No ACKD *n* (%)	ACKD *n* (%)	Total *n* (%)	*p* Value
Total	235 (79.4)	61 (20.6)	296	
*VHL*	104 (44.3)	22 (36.1)	126 (42.6)	0.314
*PBRM1*	82 (34.9)	20 (32.8)	102 (34.5)	0.875
*SETD2*	46 (19.6)	8 (13.1)	54 (18.2)	0.328
*BAP1*	25 (10.6)	1 (1.6)	26 (8.8)	0.050
*MTOR*	19 (8.1)	6 (9.8)	25 (8.4)	0.857
*ARID1A*	16 (6.8)	8 (13.1)	24 (8.1)	0.179
*KDM5C*	18 (7.7)	2 (3.3)	20 (6.8)	0.353
*FAT1*	13 (5.5)	4 (6.6)	17 (5.7)	1.000
*PIK3CA*	13 (5.5)	2 (3.3)	15 (5.1)	0.699
*TP53*	8 (3.4)	6 (9.8)	14 (4.7)	0.077
*PTEN*	11 (4.7)	2 (3.3)	13 (4.4)	0.900
*NFE2L2*	9 (3.8)	1 (1.6)	10 (3.4)	0.656
*STAG2*	9 (3.8)	1 (1.6)	10 (3.4)	0.656
*MET*	5 (2.1)	3 (4.9)	8 (2.7)	0.451
*KDM6A*	7 (3.0)	0 (0.0)	7 (2.4)	0.373
*NPNT*	6 (2.6)	1 (1.6)	7 (2.4)	1.000
*NF2*	4 (1.7)	2 (3.3)	6 (2.0)	0.788
*ELOC*	4 (1.7)	1 (1.6)	5 (1.7)	1.000
*KIT*	3 (1.3)	2 (3.3)	5 (1.7)	0.601
*SMARCB1*	2 (0.9)	3 (4.9)	5 (1.7)	0.101
*SLITRK6*	4 (1.7)	0 (0.0)	4 (1.4)	0.686
*TXNIP*	4 (1.7)	0 (0.0)	4 (1.4)	0.686
*TERT*	2 (0.9)	1 (1.6)	3 (1.0)	1.000
*RHEB*	2 (0.9)	1 (1.6)	3 (1.0)	1.000
*BTNL3*	1 (0.4)	1 (1.6)	2 (0.7)	0.878
*MSR1*	1 (0.4)	1 (1.6)	2 (0.7)	0.878
*CCNB*	0 (0.0)	1 (1.6)	1 (0.3)	0.467
*FH*	1 (0.4)	0 (0.0)	1 (0.3)	1.000
*CCND1*	1 (0.4)	0 (0.0)	1 (0.3)	1.000

**Table 3:** Univariable and multivariable analysis of *BAP1* somatic mutational status in RCC specimens.

		No	Yes	OR (Univariable)	OR (Multivariable)
ACKD	No	210 (89.4)	25 (10.6)	-	-
	Yes	60 (98.4)	1 (1.6)	0.14 (0.01–0.68, *p* = 0.056)	0.15 (0.01–0.75, *p* = 0.067)
Age at Diagnosis	Mean (SD)	60.5 (11.6)	57.5 (12.9)	0.98 (0.95–1.01, *p* = 0.225)	-
Sex	Female	93 (88.6)	12 (11.4)	-	-
	Male	177 (92.7)	14 (7.3)	0.61 (0.27–1.40, *p* = 0.237)	-
Histology	Clear Cell	170 (89.0)	21 (11.0)	-	-
	Papillary	32 (100.0)	0 (0.0)	-	-
	Chromophobe	20 (100.0)	0 (0.0)	-	-
	NOS	48 (90.6)	5 (9.4)	0.84 (0.27–2.20, *p* = 0.745)	-
Stage	I	133 (97.8)	3 (2.2)	-	-
	II	29 (93.5)	2 (6.5)	3.06 (0.39–19.25, *p* = 0.232)	3.19 (0.40–20.32, *p* = 0.217)
	III	83 (83.0)	17 (17.0)	9.08 (2.94–39.74, *p* = 0.001)	8.97 (2.89–39.43, *p* = 0.001)
	IV	25 (86.2)	4 (13.8)	7.09 (1.48–37.88, *p* = 0.014)	6.32 (1.31–33.95, *p* = 0.021)

## Data Availability

The data included in this work were obtained through the Avatar Project managed by Aster Insights under the Total Cancer Care (TCC) protocol at ORIEN Member institutions.

## Supplementary Materials

The following supporting information can be downloaded at: https://www.mdpi.com/article/10.3390/cancers16101920/s1, Table S1. Significantly mutated genes in renal cell carcinoma identified by The Cancer Genome Atlas. Table S2. Somatic mutation rates in clear cell RCC specimens stratified by presence of ACKD. Table S3. Univariable and multivariable analysis of VHL somatic mutational status in RCC specimens.
