# Supplementary material for: Clinical and Genomic Features of Patients with Renal Cell Carcinoma and Advanced Chronic Kidney Disease: Analysis of a Multi-Institutional Database
Source: Cancers (Basel). 2024 May 18;16(10):1920. doi: 10.3390/cancers16101920 (PMC11119962; doi:10.3390/cancers16101920)
Supplement: Supplementary file 1 [file cancers-16-01920-s001.zip › cancers-3001318-supplementary.pdf]

Supplementary Table S1. Significantly mutated genes in renal cell carcinoma identified by The Cancer Genome Atlas.

Histology	Significantly Mutated Genes (SMGs)
**Clear cell RCC**	*VHL, PBRM1, SETD2, KDM5C, PTEN, BAP1, MTOR, TP53, PIK3CA, MSR1, TXNIP, TCEB1, NFE2L2, BTNL3, SLITRK6, RHEB, ARID1A, NPNT, CCNB*
**Papillary RCC**	*MET*, *SETD2*, *NF2*, *KDM6A*, *SMARCB1*, *FAT1*, *BAP1*, *PBRM1*, *STAG2*, *NFE2L2*, *TP53*, *FH*, *NF2*
**Chromophobe RCC**	*TP53, PTEN, TERT, KIT*
**Oncocytoma**	*CCND1*

Supplementary Table S2. Somatic mutation rates in clear cell RCC specimens stratified by presence of ACKD.

Mutated Gene	No ACKD *n* (%)	ACKD *n* (%)	Total *n* (%)	p value
Total	156 (81.7)	35 (18.3)	191	
VHL	82 (52.6)	16 (45.7)	98 (51.3)	0.575
PBRM1	65 (41.7)	12 (34.3)	77 (40.3)	0.452
SETD2	41 (26.3)	6 (17.1)	47 (24.6)	0.287
KDM5C	15 (9.6)	2 (5.7)	17 (8.9)	0.743
PTEN	7 (4.5)	1 (2.9)	8 (4.2)	1.000
BAP1	20 (12.8)	1 (2.9)	21 (11.0)	0.132
MTOR	18 (11.5)	4 (11.4)	22 (11.5)	1.000
TP53	4 (2.6)	3 (8.6)	7 (3.7)	0.117
PIK3CA	11 (7.1)	1 (2.9)	12 (6.3)	0.699
MSR1	1 (0.6)	0 (0.0)	1 (0.5)	1.000
TXNIP	4 (2.6)	0 (0.0)	4 (2.1)	1.000
ELOC	3 (1.9)	1 (2.9)	4 (2.1)	0.558
NFE2L2	8 (5.1)	0 (0.0)	8 (4.2)	0.355
BTNL3	1 (0.6)	0 (0.0)	1 (0.5)	1.000
SLITRK6	2 (1.3)	0 (0.0)	2 (1.0)	1.000
RHEB	2 (1.3)	1 (2.9)	2 (1.0)	0.457
ARID1A	13 (8.3)	5 (14.3)	18 (9.4)	0.333
NPNT	4 (2.6)	1 (2.9)	5 (2.6)	1.000
CCNB1	0 (0.0)	1 (2.9)	1 (0.5)	0.183
MET	1 (0.6)	1 (2.9)	2 (1.0)	0.334
NF2	3 (1.9)	0 (0.0)	3 (1.6)	1.000
KDM6A	5 (3.2)	0 (0.0)	5 (2.6)	0.587
SMARCB1	1 (0.6)	1 (2.9)	2 (1.0)	0.334
FAT1	11 (7.1)	3 (8.6)	14 (7.3)	0.724

STAG2	4 (2.6)	1 (2.9)	5 (2.6)	1.000
FH	1 (0.6)	0 (0.0)	1 (0.5)	1.000
TERT	1 (0.6)	0 (0.0)	1 (0.5)	1.000
KIT	1 (0.6)	1 (2.9)	2 (1.0)	0.334
CCND1	0 (0.0)	0 (0.0)	0 (0.0)	1.000

Supplementary Table S3. Univariable and multivariable analysis of *VHL* somatic mutational status in RCC specimens.

		No	Yes	OR (univariable)	OR (multivariable)
**AKD**	No	131 (55.7)	104 (44.3)	-	-
	Yes	39 (63.9)	22 (36.1)	0.71 (0.39-1.26, p=0.250)	0.76 (0.41-1.39, p=0.371)
**Age at Diagnosis**	Mean (SD)	60.2 (12.2)	60.2 (11.2)	1.00 (0.98-1.02, p=0.975)	-
**Sex**	Female	59 (56.2)	46 (43.8)	-	-
	Male	111 (58.1)	80 (41.9)	0.92 (0.57-1.50, p=0.749)	-
**Histology**	Clear Cell	93 (48.7)	98 (51.3)	-	-
	Papillary	27 (84.4)	5 (15.6)	0.18 (0.06-0.44, p=0.001)	0.18 (0.06-0.46, p=0.001)
	Chromophobe	20 (100.0)	0 (0.0)	-	-
	NOS	30 (56.6)	23 (43.4)	0.73 (0.39-1.34, p=0.309)	0.74 (0.40-1.36, p=0.327)
**Stage**	I	84 (61.8)	52 (38.2)	-	-
	II	14 (45.2)	17 (54.8)	1.96 (0.89-4.37, p=0.094)	-
	III	57 (57.0)	43 (43.0)	1.22 (0.72-2.06, p=0.461)	-
	IV	15 (51.7)	14 (48.3)	1.51 (0.67-3.39, p=0.318)	-
